# Employee Social Network Strategies: Implications for Firm Strategies and Performance in Future Organizations

**DOI:** 10.3389/fpsyg.2021.726606

**Published:** 2021-12-13

**Authors:** Monica Thiel

**Affiliations:** School of International Studies, University of International Business and Economics, Beijing, China

**Keywords:** strategy, group entitativity, competition, organizational performance, employee social networks

## Abstract

Employee social network strategies play a key role in firm strategies and organizational performance. Currently, scholars underestimate the contributions of employee social strategies in firm strategies. Little is known how informal employee social networks, group entitativity and competition could shape and direct firm strategies and organizational performance. The article examines social network theory and strategic management’s content, process and open schools of thought to propose a new interpretation for managing firm strategies. More specifically, the author examines alternate causal paths, underlying processes and structures as mechanisms in employee social network strategies within a theoretical framework. The article proposes 4 theoretically driven propositions and makes two contributions. First, the article contributes to organizational behavior literature by focusing on the literature gap in network dynamics and competitive actions through employee social networks. Second, although there is immense literature on positive and negative employee competition in business, the article makes a contribution to the strategic management literature by moving beyond formalized structures and roles within an organization to focus on the multilevel informal workplace social interactions and processes that impact strategizing activities. Overall, the article extends strategy research in relation to how employee social networks operate through competition and group entitativity in firm strategies.

## Introduction

Employee social networks warrant significant consideration in firm strategies and performance. Previous research shows sparse literature and theory on the “network dynamics of competitive action” and “competitive behavior” (Swab and Johnson, 2018, p. 157). The article contributes to prior literature and theories on social networks to reveal competition is a process of securing productive relationships (Burt, 1992), rather than merely being a player in competitive advantage and strategic positioning between firms, industries, states, and countries (Porter, 1980). Social context plays an increasingly important role in a firm’s strategy formulation and implementation because competitiveness is often associated with and evaluated by social aspects (Thiel, 2017). Moreover, economic action is driven by social networks and relationships in a firm (Gulati, 1998). Firms are not paying adequate attention to employee social structures and social network strategies because unquantified knowledge and information is often dismissed by leadership for objective and quantifiable knowledge and information to govern an organization (Michaud, 2014). Moreover, analyzing competition is difficult due to invisible social structural holes (Burt, 1992) in employee social networks that are not directly observed by employees in a firm or within interorganizational networks and market competition. Therefore, serious and more robust employee evaluation in a firm’s social structures, organizational structures and network structures is warranted from human resource management to gage positive and negative behaviors in organizations, especially through hybrid office structures and increasing network platforms due to COVID-19. New key strategic roles are necessary for human resource, marketing and information technology departments that can partner with each department manager in the firm and externally with other firms and organizations.

The article aims to challenge strategic management literature assumptions by moving beyond a focus on organizational resources and firms’ environments as given and detached entities within firms’ relationships and quasi-universal fixed causal laws that apply across time and space (Rabetino et al., 2021). Moreover, the theoretical development in the article challenges key assumptions (Alvesson and Sandberg, 2011; Cornelissen et al., 2021) in firm strategies and deviates (Höllerer et al., 2020) from the existing strategic management literature toward continual turbulent transformation in the social environment (Teece, 2020) that has not been adequately addressed within firm strategies. Hence, scholars and practitioners underestimate the contributions of employee social strategies in firm strategies. Through a literature review, the article introduces a new interpretation for understanding firm strategies through “configurational theorizing” (Cornelissen et al., 2021, p. 7) and 4 theoretically driven proportions. The article is motivated by a cross-disciplinary approach of organizational behavior literature and strategic management literature to examine how employee negative behavior operates through relational and governance structures within the firm to reshape and redirect firm strategies. For instance, Zhong and Robinson (2021) research findings indicate that misbehaving or negative behavior has the potential to incur employee resource gains and positive results such gaining control over others, that in turn could decrease organizational performance.

There is scant literature in strategic management that examines the problem of negative individual and social behavior through employee social networks in firm strategies. Rather than a focus on social networks and *market competition*, the article examines *social competition* and the use of informal network ties to manage employee relations that span from the firm to the market. Employees do not leave their individual and collective interests at home apart from work. Rather, individual and self interests are part of the relational and governance structure in firms and are a strategic mechanism for managing who gets hired, promoted and governed for social reputation, resource constraints and social/professional status. For instance, Westphal et al. (2006) propose “despite limited prior evidence that resource dependence determines the formation of formal board ties, corporate leaders may nevertheless reconstitute informal (i.e., friendship) ties to leaders of other firms that have the power to constrain their firms’ access to needed resources when those ties have been disrupted (e.g., due to turnover of the CEO’s friend)” (p. 425). The article makes two contributions. First, the article contributes to the organizational behavior literature by focusing on the largely unexplored network dynamics of competitive action (Swab and Johnson, 2018, p. 157) through employee social networks, competition and group entitativity. Second, although there is immense literature on positive and negative employee competition in business, the article makes a contribution to the strategic management literature by moving beyond formalized structures and roles within an organization to focus on the multilevel informal workplace social interactions (Winslow et al., 2019), “alternate causal paths” and “underlying processes and structures as mechanisms” (Cornelissen et al., 2021, p. 7) that impact strategizing activities. Hence, the article extends strategy research in relation to how employee social networks operate through group entitativity in firm strategies. Entitativity is defined as pure group solidarity (Campbell, 1958) through physical, goal, behavior, similarity, and extent of interactions (Lickel et al., 2000). Entitativity operates through a cohesive group that shares static traits such as appearance in ethnicity and background in education and dynamic traits such as goals (Campbell, 1958). Therefore, the behavior of a high entitativity group’s perception of group members will most likely align with the group’s goals (Gergen et al., 1973) when the group shares similar appearance, behavior and outcomes. Social network theory in organizations suggests formal and informal social relationships form positive, neutral (supportive) and negative ties or relationships (Marineaua et al., 2018). Hence, employee social networks often operate within multiplex networks that are defined as signed networks or signed graphs (Harrigan et al., 2020).

Definitions and concepts of competition and competitiveness vary based on different research frameworks (Swab and Johnson, 2018). Individual and social competition often operates within absorptive capacity (Zou et al., 2018). The article defines individual and social competition as personal and collective competitiveness to undermine and win control over others that do not share the same behavior similarity, interests, values, and goals. Although, competitive interactions between employees could be decreased and prevented through trust and cooperation, the definitional assumption of competition fails to recognize how group entitativity could foster and increase competitive interactions between employees and increase employee invisibility behavioral practices (Anteby and Chan, 2018) through network structure cooperation and fragmentation. The article begins with a discussion of relevant strategic management literature, employee social networks, a theoretical framework, followed by a discussion, main limitations and future research with concluding comments.

## Strategic Management

Strategic management approaches are highly dependent on the changing environment (McGrath, 2013). Molina-Azorin (2014) suggests the literature in the knowledge-based view of the firm has been dominated by a macro orientation that considers constructs at the level of the firm rather than the skills, efforts, knowledge and behaviors of individuals operating within rapidly changing uncertain environments. Moreover, theories of strategy and organization often depict organizations as unitary actors, rather than collections of individuals (Felin and Zenger, 2009). On the contrary, individual routines play an integral role in how a firm competes (Barnard, 1938; Aime et al., 2010). Despite disagreement among some scholars about the role of the individual to explain phenomena on a micro-level (Hodgson, 2012), social regularities vary and change, and require consistent re-evaluation of how individual order shapes and drives social order, especially within the failure of “economics imperialism” (King, 2012).

Previous research indicates that employees could become disloyal and resistant to an organization’s identity and pursue identity-inconsistent strategies (Ravasi and Phillips, 2011) that in turn redirect firm strategies through an “identity–strategy misalignment” (Wenzel et al., 2020, p. 212). Moreover, employee coping strategies and tactics could dismantle firm strategies through misalignment of firm strategies through revised employee strategies. For instance, management could pursue market-oriented strategies to remain competitive rather than the firm’s technology-focused identity and strategies (Nag et al., 2007). Examining the micro-level analysis of a firm’s strategies and performance provide revitalization to bridge strategic management’s theory-practice gap where social systems, scientific knowledge and professional practice operate interdependently (Dobusch and Kapeller, 2013; Cornelissen and Durand, 2014; Fisher and Aguinis, 2017; Drnevich et al., 2020). Furthermore, Lehmann-Willenbrock and Allen (2018) propose actual behavior is significantly understudied in psychology, despite psychology’s scientific aim to explain human behavior because psychology often provides scientific evidence through actual behavior and temporal interaction data within student samples. Therefore, examining behavioral micro-processes in the real world of firm strategies could help to improve and advance understanding of actual temporal interaction data and analyses that occur within organizations and shape and drive employee social interactions and competitive actions in networks over time. Firm growth could be usefully studied as a social dynamic process (Pisano, 2016) of management interacting with resources. However, the firm level as a driver for firm growth, competitive advantage and collective productive resources prevents the firm from achieving stronger individual and organizational performance because the firm level ignores and minimizes the capacity and resources that individuals possess to shape and redirect the firm’s resources and capabilities. Individuals could act *ad hoc* and irrational within different contexts. Hence, the determinants of firm performance require dynamic and static analysis at the individual level. For instance, Arain et al. (2018) found that knowledge hiding can spread from supervisors to subordinates.

Competitive analysis approaches in strategic management analyze major forces acting on an industry, such as the power of buyers and suppliers, the prospects for substitute products, and competition in its markets (Porter, 1985). Firms establish strategies to gain competitive advantage over their competitors through differentiation and selecting the segments of an industry in which a firm should compete (Porter, 1998). Content placed strategy making in planning (Ansoff, 1965) and positioning (Porter, 1980) traditionally focused on top management to formulate and implement strategies secretly through a macro-level perspective. Strategy process opened the door to a more comprehensive strategic plan of beliefs, goals and priorities (Dobusch and Kapeller, 2013) that is shared with select stakeholders actively participating in the strategy making process (Yunus et al., 2010; Castells, 2015). Process strategy has led to more open strategies (Whittington et al., 2011) due to increasing transparency and participation across sectors and industries with inclusion and collaboration of stakeholders in strategy practice.

Content and process schools reinforce the micro-macro link between networks, firm strategy and performance. However, both schools permit employees to continually operate informally through employee interactive networks that reshape firm strategies with select stakeholder networks that redirect firm performance in market competition. Open strategies that emphasize transparency and inclusion within open practices require careful consideration managing dilemmas on the organizational level and individual level (Hautz et al., 2017) due to employee social networking strategies. Inclusion of employees through the active process of strategy making, commenting and evaluation of ideas offers (a) opportunities for employee social networks to decrease or disregard organizational transparency and inclusion and (b) opens the door to increase the opportunities for employee social network strategies rather than merely relying on firm strategies. Overall, strategy making through content, process and open schools of thought is a social process (Hautz, 2017). Due to strategic management’s value and significance to improve organizational performance, it should not be surprising that individual and social competition is easily embedded into the social processes of strategy making. Neither, is it surprising that firm strategies could be undermined by individual employees prominent in social networks, but are invisible to corporate hierarchy (Burt and Ronchi, 1990).

## Employee Social Networks

### Real World Networks

Networks of interconnected organizations and networks of individuals, leadership and teams will be managed in future organizations within “complex systems that produce chaotic outcomes such as emergent properties that are prone to large changes in outcome as a result of small changes in the relevant variables” (Teece, 2018, p. 362). Therefore, current and future organizations that operate in high uncertainty and constant change require a more “granular level of analysis that allows organizations to tap into the informal communication networks that determine how work in organizations really gets done” (Eisenberg et al., 2015, p. 152). Clearly, employee social network strategies have a strong potential to become deeply embedded within an organizational culture through sub-groups that act against firm-wide rules with or against others’ consent (Thiel, 2020). Moreover, authority in an organization does not follow merely from a leadership position because a simple diagram of an organization do not show the full activity of all the leaders. Often, the most influential employees are those with the least formal authority that may govern an organization autonomously. For instance, an employee can take the vision and idea from another co-worker or leader and publicly depict the vision and idea as the employee’s original conception. Hence, Ghawi and Pfefer (2022) propose “The increasing need for handling real-world networks requires a deeper investigation of a multilayer network” (p. 1). Organizational network research in informal employee networks is challenging. There are an increasing number of scholars searching social networks in business and management settings (Cronin et al., 2021). However, real-world networks are constrained due to employees’ concerns of privacy, sensitive issues, job security and management impact of employees’ relationships that are considered outside of management purview.

### Organizational and Social Structure in Employee Social Networks

Organizational structures are shaped through individual and social competition that operate within social interactions between individuals. Consequently, firms require shifting human capital to a more equal footing with financial capital for managing and improving organizational performance rather than disregarding the employee social processes that drive organizational performance. Moreover, the emphasis on financial capital to drive organizational performance permits wide gaps for employee discretion of employee valuation and abuse in the workplace. Hence, performance is a continually evolving social interactive process and construct (Thomas, 2006).

The continual emergence of new technologies require continual reassessment of firm strategies and employee social network strategies in changing organizational structures such as platforms. Cennamo (2016) proposes “platform value and ecosystem structures co-evolve *via* complex feedback effects” (p. 3061). Moreover, the structure of ecosystems-based platforms “explicitly extends the strategic view to include activities and actors over which the focal organization may have no control, and with whom they have no direct contact” (Adner, 2017, p. 44). Thus, individual and social competition can drive or constrain value creation-capture dynamics within and across competing ecosystems. This requires changing the way organizations examine and understand how employee social networks drive the value-creation process. In addition, platforms foster innovation and efficiency across diverse sectors requiring coordination, strategy and performance integration across organizational units and firms within conflicting interests or requirements. Therefore, competitive and cooperative behavior in organizational platform structures necessitate re-evaluating broader sets of capabilities and processes for redesigning market competition within parts of one organization to another organization, especially in asymmetric and hierarchical forms of organizing that do not disappear in new multinational network forms (Clegg et al., 2018).

There is growing emphasis from scholars on new collaborations across sectors and industries in responsible innovation (Owen et al., 2012) and open innovation. Digital platforms and interactions with artificial intelligence can change the way employees collaborate and compete through autonomous behavior in social network dynamics, making strategy formulation and implementation more challenging. In addition, human capital is not homogenous (Barney and Felin, 2013; Ostroff and Bowen, 2016). Therefore, the focus should be on capturing adaptation and value rather than merely static forms of organizing for determining individual and group preferences over others (Thiel, 2016). The rise of decentralized hybrid teams working through business innovation that reshapes the authority of an organization’s structure and generates an employee network culture have access to a wider variety of skills and people, making dissemination of disinformation about individual employees and individual firms more likely to occur. Therefore, firms must (a) become strategically decisive to monitor and control employee social networks and their influence with competitors and (b) develop organizational strategies and performance internally through its employees, rather than poach from other firms to develop competitive capabilities. Hence, formulating and implementing strategies in organizations require examining systemic employee social networks with formal (Graen and Cashman, 1975) and informal planning systems for improved strategic control.

Although most information exchanges and mobility events may occur at short socio-metric distances (Sin et al., 2009; Othman et al., 2010; Dulebohn et al., 2012; Joseph et al., 2015; Sheer, 2015; Liao et al., 2017) the article focuses on short, medium and long-range structures located in the network as a whole. Employee social networks consist of both positive and negative reciprocated and non-reciprocated ties (Pauksztat and Salin, 2020). It is not uncommon for employees to enact “invisibility strategies” or “coercive surveillance” to resist workplace surveillance (Anteby and Chan, 2018, p. 1 and p. 13). Thiel et al. (2019) suggest it is important to examine an organization’s culture with social structure linkages consisting of individual and social competition between the firm, within the firm, and connections through stakeholder interactions because employee social network strategies derive from relational networks, and human capital and cognition (Oliveira Correa et al., 2018). Many firms do not evaluate employee social networks and may be blind to where the most critical employees sit within a network. Hence, there is an unnoticed capacity (Holz and Miller, 2001; Kellogg, 2009; Kelly, 2014) from management based on how employee social networks control firm strategies. This is important because employee networks could work to downgrade another employee’s skills and knowledge for personal gain. For instance, employees working and living in geographic areas with high corruption and high risk communities connect with their community peers that are working and living in low corrupt and low risk geographical areas. The employees and community peers monitor and control the work relations, social identity and reputation of community outsiders that become aware and learn how informal deviant behavior is sustained from the employees working and living in geographic areas with high corruption and high risk communities and from community peers that are working and living in low corrupt and low risk geographical areas. Moreover, since employee networks operate across networked organizations, social control is easy for employees collaborating with community peers because they could persuade human resources staff and leadership (Liden and Antonakis, 2009) to accept their evaluation of an employee that previously was a community outsider. Hence, employee networks are not confined to the business environment. Rather, employee networks generate nodes within society at large that connect into firm networks and ecosystems.

### Levels of Analysis in Employee Social Networks

**FIGURE 1:**
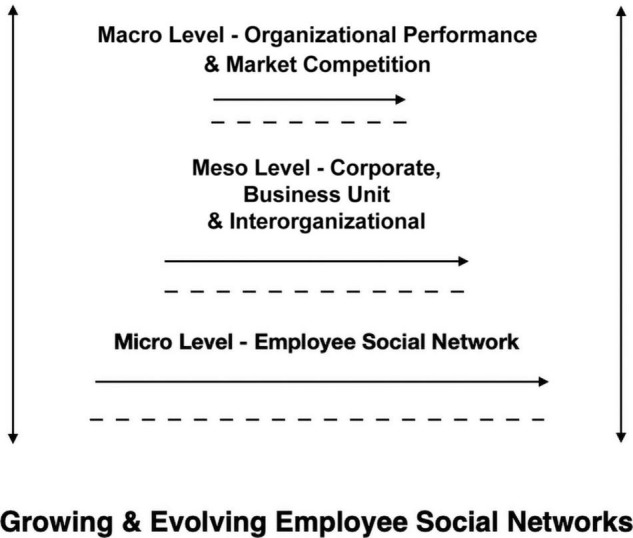
Levels of analysis.

Employee social network strategies operate through three levels of analysis (micro, meso and macro). Figure 1 highlights the connected micro-meso-macro levels beginning with the foundation of employee social networks at the micro level, corporate, business unit, and interorganizational strategies at the meso level and organizational performance and market competition at the macro level. Employee social networks derive from informal individual employee strategies that evolve and merge with formal firm strategies through the connected micro-meso-macro levels and in turn, could improve or decrease organizational performance.

Employee social networks consist of strategic interactions that form multilevel networks and intertwine with existing networks worldwide to secure individual and collective interests within the foundation of an institution, a community and its ecosystem. Employees communicate within centralized, decentralized, and distributed networks (Vergne, 2020). Consequently, network multiplicity occurs within multilevel employee social networks that share market knowledge and employee reputations to industry stakeholders operating and communicating with market actors in market competition.

**FIGURE 2:**
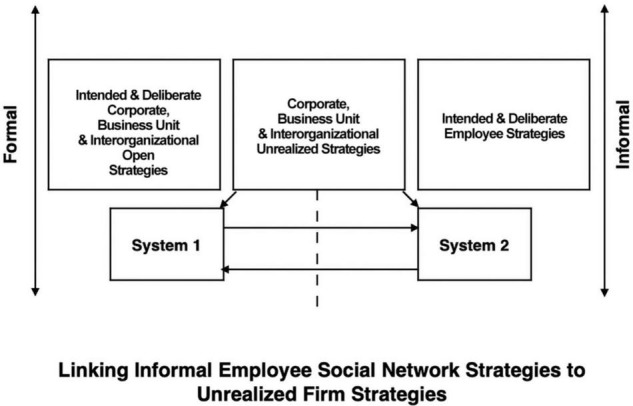
Formal firm-informal employee interface.

Mirabeau et al. (2018) identify six manifestations of strategy namely, intended, deliberate, unrealized, realized, emergent, and ephemeral strategies. Informal employee social network strategies evolve and grow from individual and social competition and reconfigure the network paths of formal firm intended and deliberate corporate, business unit and interorganizational open strategies. Employee informal intended and deliberate social strategies could thwart a firm’s formal strategies into unrealized strategies (Figure 2). Consequently, informal employee intended and deliberate strategies may be considered merely insignificant ephemeral strategies, but in fact are constructed social strategies (Suominen and Mantere, 2010). It is important to highlight the strong potential of employee autonomous behavior that could work to reconfigure effective firm performance. For instance, a CEO’s unsuccessful visionary strategy may implicate the cause to poor emergent firm strategies rather than informal employee strategies within the organization. Moreover, Maritz et al. (2011) findings indicate “emergent strategy making is associated with quick response and adaptation to environmental changes, more autonomous decisions and actions, less control and higher intangibility” (p. 101). Consequently, employees could have multiple simultaneous strategies that coexist and align with a CEO’s strategy.

## Theoretical Framework

### Individual and Social Competition Typology

Employees strategize through day-to-day activities (Jarzabkowski, 2005) and day-to-day relationships. Competition is not always about winning or assessing how an individual employee compares to her or his peers. Rather, competition is dynamically shaped through strategic interests and interactions within networks. Bilancini et al. (2019) suggest competition often involves strategic interactions such as a strategic quotient test to determine abilities and rationality for strategic success. Strategic interactions are difficult to measure because it is a deviant form of behavior in that it takes place without organizational approval. Evidence provided by Bilancini et al. (2019) collected data indicate success is dependent on understanding of others’ preferences and understanding of others’ cognitive skills. In addition, Thye et al. (2011) found evidence for competitive networks that are structurally more cohesive tend to “promote group formation among self-interested actors who pursue those interests through dyadic exchanges” (p. 409). Thus, individual and social competition is often driven by a mechanism of mentalization (Wang et al., 2018) in strategic interactions. The authors, Wang et al. (2018) propose two new psychological measures namely, competitive attitude and competitive behavior to show competitive behavior is driven by some internal psychological characteristics that can be changeable and adaptable under different environments. Moreover, the authors’ findings indicate cooperation can coexist with other strategies (Deutsch, 1949; Nowak, 2006; Krueger, 2013; Gnyawali and Charleton, 2018).

The typology shows the potential antecedents that initiates informal employee strategic interactions through self, shared, collective, or relational interests. For instance, a new employee may be perplexed on whether to listen and follow a supervisor exclusively regarding beliefs and values. In turn, beliefs and values may often play a role on whether the supervisor will like an employee or not (Blair et al., 2017). If the new employee maintains a neutral position or favors the values and beliefs of another employee rather than the supervisor, the staff will work more closely together informally, rather than formally with the supervisor and other employees. Hence, proposition 1 proposes informal employee social network strategies are driven by strategic behavior and interactions within individual and social competition. Individuals make latent decisions about employees that begin from individual and social competition and widen in scope through multilevel social networks. Moreover, these social networks often work in protective ways to ensure that employees are selected and promoted according to the individual and social interests of the network. Therefore, informal employee individual and social competition could easily reconfigure firm strategies and performance.

Individual and social competition in Table 1 specifies the initial conditions for group entitativity to develop and grow. The typology begins with personal self-interest followed by shared self-interest, collective self-interest, and lastly relational self-interest. The interest type characteristics indicate increasing satiation of interests that move from the micro level to the meso level and onto the macro level. Individual and social competition could be utilized within person-to-person or organization-organization interactions. In the typology, value related factors (Felin et al., 2015) are mechanisms for self, shared, collective and relational interests that aggregate, develop, and grow within informal employee socialization in the organization and externally with stakeholders and market competition. For instance, power in Table 1 could be represented in both formal and informal self-interest forms. Formal power could be exercised through the leadership in a firm as formal routine tasks. Informal power occurs within social and cultural interactions between individuals and organizations and how they relate to each other (Huxham and Beech, 2008). Marineaua et al. (2018) research study findings indicate “individuals with power can indeed be more accurate about social network ties; however, we also found that when the person with power is directly involved, accuracy increases” (p. 156).

**TABLE 1:** Typology of individual and social competition.

Interest type	Self interest	Shared interest	Collective interest	Relational interest
**Value factors**	Personal choice; self-attributed traits; self-worth; emotional satisfaction; individual comparison; reputation; knowledge; skill development; freedom; indifference; age; gender; first place; power	Social purpose/cause	Pre-determine others; social control; social comparison; social evaluation; similarity of traits and values; social identity; social interaction rules/norms; cooperation; identity governance	Specific friends and family members

### Group Entitativity

Group entitativity is a mechanism in network formation and in the selection of relational and governance structural processes that encompass “reciprocity,” “popularity,” “activity,” “triad closure,” and “brokerage” “endogenous structural processes” (Kim et al., 2016, p. 25) in varied and combined network structures. It is important to examine group entitativity because employees’ can easily obtain social control in a firm through employee autonomy such as generating open and innovative strategies, and problem solving (Hautz et al., 2017). Entitative groups form a coherent entity. Groups perceived with significant entitativity through high degrees of interactions and goals will become more intimate (Vock et al., 2013) and may provide greater need fulfillment than less intimate groups (Crawford and Salaman, 2012). Group entitativity is driven through focused impression management of individual and group efforts because employees are treated by others in the organization and within society at large in terms of both their shared individual qualities and their group affiliations for work and social status. Adelman et al. (2018) propose “perceptions of entitativity may also be influenced by motivation and are adapted in self-serving ways to create the necessary conditions for holding other groups responsible or not” (p. 37). Group entitativity is appropriate for determining useful predictions that are sustainable and forward looking causal mechanisms rather than merely relying on professional experience (Anand et al., 2016) as a source of a firm’s capability because employees frequently makes implicit decisions based on personal and collective interests through social networks in the workplace. Hence, proposition 2 suggests individual and social competition develops and grows group entitativity.

Managing the true impacts of a business can improve performance and competitiveness, making it better and less costly for a firm than doing nothing (Griffin and Freeman, 2016). Clearly, who contributes to strategy variation, selection and retention (Arndt and Norbert, 2015) as well as who realizes when employees such as leaders and subordinates unite in group entitativity requires rethinking employees’ discretionary impacts in firm strategies and performance because group entitativity is easily hidden in firm strategies and performance. Previous research in entitativity suggests it is common for individuals to describe organizations as separate entities (Koivunen, 2009) and foster group-like thinking and practices (van Vuuren et al., 2012). Within social competition, outgroup entitativity increases fear and negative responses from the entitative group because a competent out-group opponent is more dangerous than an incompetent out-group opponent (Frauen et al., 2020). Stollberg et al. (2015) research study indicates “some groups are better suited to fulfill a need for control than others: when multiple in-groups are salient in a situation, people respond to control threat by increasing identification only with those groups that are both highly entitative and agentic” (p. 10). Likewise, Insko et al. (2013) empirical research findings indicate

fear and greed flow from perceptions of entitativity, and entitativity perceptions, in turn, are strongly influenced by the group’s decision-making rule— whether group members’ choices are aggregated following a simple majority rule. On the basis of these results, we would advise managers and employees to be mindful of the decision rules they employ in organizational settings. Inter-departmental cooperation within organizations can be undermined by decision-making rules, like majority-vote, that increase greed within a particular group (or department) and decrease trust from other groups (p. 179).

Group types vary in level of perceived entitativity (Lickel et al., 2000). Employees may actively seek to cooperate with other members for benefits from knowledge sharing (Lee and Yang, 2014). However, group entitativity displays a unified entity that flourishes through individual and collective interests. Moreover, patterns of group entitativity can rise and fall. Hence, weak employee social network ties do not imply group entitativity is absent. Empirical evidence reveals entitativity increases with group size and decreases with variability and diversity due to how meaningful a stimulus pattern is (McCarthy et al., 1995). Group entitativity is significant in shaping the social identity of members and the internalization of group norms such as shared norms, mutual acceptance, attraction to the group and the resistance to disruptive influences through psychological processes (Hogg and Reid, 2006). It is a form of authoritarian governance that is strategic through prospering as an organic process, rather than through command and control. In addition, complex social environments could make employee social networks invisible in an organization. For instance, employee social networks can hide informal practices through dispersed teams operating in network infrastructures and swarm work because the employees are unlikely to charge each other.

Personal and group interests and values form the strategic competitive actions through employee interactions in social networks. Firat et al. (2018) suggest “value priorities act as more than a personal moral compass; they constitute the basis of shared group moral understanding” that create a bond for group entitativity (p. 1). One example of group entitativity is Chinese guanxi’s social networks and family relationships that take precedence and preference over other individuals and groups. A second example is networked cartels, organizations and communities that work to contain and monitor transformation threats with individual employees in networked organizations and markets across the globe. A third example is entitative group members in a “platform ecosystem can influence the behavior and outcomes of other members and the outcomes for the ecosystem overall” (Rietveld and Schilling, 2020, p. 24) in positive and devious ways. For instance, “despite the ability of networks to provide opportunity, networks can also close opportunities or reproduce inequities in employment access” (Jabbar et al., 2020, p. 1489). Moreover, employees may be ignorant and unaware of group entitativity operating in a firm because there may be merely a few individual employees that connect with employees from other networked organizations that form group entitativity. Hence, proposition 3 proposes group entitativity fosters protective employee interorganizational network interactions that span across industry stakeholder connections and market competition.

### Interlocking Personal and Work Ties as Interorganizational Networks

The social process and network formation could easily begin with “prior interlocking ties” (Kim et al., 2016, p. 31) from an employee working in a firm that connects with known external actors to make new ties within interorganizational networks. An entitative group could be culturally normalized through networked employees, organizations and markets. The group forms through employees’ social network of workplace rules, how tasks are done, and employee skill and value expectations within interorganizational networks. For instance, an individual interviews for a position with the marketing director of a firm. The interviewee finds the marketing director questioning why the interviewee is not interacting and behaving in the way the director was informed by the marketing director’s interorganizational social networks where the interviewee previously worked. The marketing director’s interorganizational social networks depicted the interviewee as having negative social interactions to downgrade the interviewee’s future employability and undermine the interviewee’s ability and skills to succeed in future employment. However, the interviewee is interacting naturally with the marketing director during the interview. The interviewee follows up with the marketing director about the position. The marketing director hired someone else. The example shows the marketing director is an external employee social network member with the interviewees’ previous employer. The internal employee social networks know the marketing director because the marketing director is from the same ethnic group. It is common for employees to form social networks through group entitativity based on ethnicity or physical appearance (Lickel et al., 2000) to protect and stick together for collective interests. The marketing director was given false information about the interviewee because the interviewee resigned from the previous position due to hidden unethical business practices. The marketing director could be unaware of the hidden unethical business practices that are occurring within the network members or the marketing director is aware of the unethical practices and is simply protecting the concerns and issues of outsiders from network members. Nevertheless, group entitativity is formed through the internal and external employee networks’ social control in both firms. The interorganizational impacts operate through informal and formal “density, paths, reciprocity, activity, and popularity spreads” (Zappa and Lomi, 2015, p. 555) across sectors, industries, and stakeholders that connect into market competition. Hence, interorganizational networks have strong potential to decrease employee value creation and organizational performance. Interlocking networks tend to take similar stances on a wide variety of issues that overlap with other forms of information and resource sharing (Messamore, 2021). Since employee networks often lead toward groups of mutual exclusivity in organizations, it will be a complex challenge to determine the key network actors. Therefore, managers should take advantage of the firm’s embedded social relations and social structures (Wolff et al., 2021) to determine the direction of the firm’s strategies and to better evaluate and measure organizational performance.

Informal social status plays a key role on how an employee is treated by the actors in the organization. Low social status in a network provides benefits in the networks and little cost to an employee that spreads negative information about another employee (Ellwardt et al., 2012). By focusing on negative influence to decrease the social status of an employee in an organization, personal and work ties within interorganizational networks are likely to accept the spreading of the negative influence and information on the employee, including reinforcing the belief among internal and external network ties that the employee deserves low social status. Employee social networks flow firm resources and capabilities to make judgments about each actor’s status within the organization and externally to other organizations. Although network nodes and links in the real world are embedded in a physical space, whereby the interactions between the nodes depend on the geometrical distance between nodes (Braha et al., 2011), the theoretical framework is addressing employee social networks that are not dependent on the network physical space, but rather the preference of members through group entitiativity and within the initiating conditions of individual and social competition in the typology.

**FIGURE 3:**
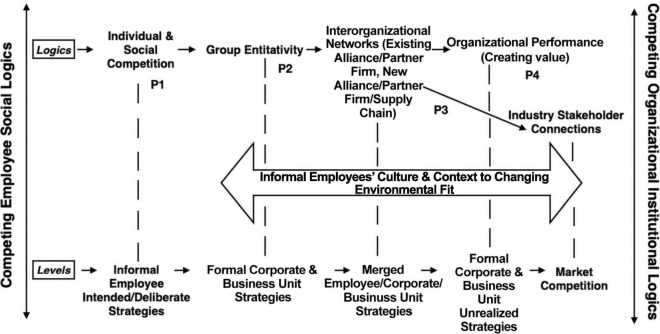
Connecting informal employee social network strategies to formal firm strategies.

The predominant approach to studying social networks assumes that the network exists independent of each actor (Marineaua et al., 2018). However, in this article the network is not independent of each actor due to the embeddedness of group entitativity. Hence, the signed graph network (Harrigan et al., 2020) is formed and sustained through group entitativity. Group entitativity is a mechanism that gives the employees strategic advantage over other employees within the organization. The employees influence other actors within the firm and across organizations and communities. In Figure 3, individual and social competition specifies the initial conditions in employee social networks.

### Step-by-Step Process of Connecting Informal Employee Social Network Strategies to Formal Firm Strategies

Employees located at the corporate level (remote/industry) engage in informal self, shared, collective and/or relational interest that join group entitativity with employees at the formal business unit (remote, industry, market) levels. The informal employee networks merge formal corporate and business unit strategies within interorganizational networks and industry stakeholder connections in market competition at the community, regional, national, and global levels. In turn, unrealized formal corporate and business unit strategies and decreasing organizational performance flourish. In addition, it is important to pay attention to how informal employee networks’ culture and context could change the strategic activities’ environmental fit as well. Overall, Figure 3 reveals how closed homogenous network ties work through open heterogenous network ties to reconfigure different levels of strategies from different involvement of employee networks.

### General Background Summary of Connecting Informal Employee Social Network Strategies to Formal Firm Strategies

Utilizing configurational theorizing (Cornelissen et al., 2021, p. 7), employees are tangible assets that generate multilevel informal workplace social interactions (Winslow et al., 2019) through “alternative causal paths” (Cornelissen et al., 2021, p. 7) such as positioning the marketplace of firms within unproductive relationships and prevent partnering with other successful firms. Alternate causal paths operate through informal employee social interactions within group entitativity as the mechanism and the firm’s resources and capabilities as an inviting structure for group entitativity to flourish within the firm and externally to other organizations as well. Group entitativity within employee networks could produce differing social interactions due to local and national cultural factors such as individualism, collectivism, masculinity, work attitudes, status, time orientation, power distance (strict/flexible boss-subordinate relationships), and face-saving. Attention to contextual cultural factors operating within group entitativity is important due to how group cultures may be obscured within the formal organizational culture. Contextual factors such as national, state, local, and organizational policies, community norms, practice culture, legal environment, historical factors, and recent events may change employee network group entitativity over time. Furthermore, employee networks could easily transcend national boundaries into global communities that are obscure to firm strategy activities. Overall, a parent company that directs other subsidiaries located in different countries require contextual and cultural views of group entitativity within employee networks. The “underlying processes and structures as mechanisms” (Cornelissen et al., 2021, p. 7) include individual and social competition, and group entitativity. Hence, informal employee network strategies shape and redirect a firm’s core competencies and capabilities, which in turn impacts the firm’s strategic activities, environmental fit, market competition and ability to create value through organizational performance.

Group entitativity plays a key role within interorganizational networks. The personal and work tie interlocks in the logics and levels act as pipes that spread information through unequal opportunity structures (Heemskerk, 2013) within organizations and position key network actors’ negative and positive influence across dyads and within internal and external network ties. Informal employee strategic interactions between internal employee networks and external employee networks become formally integrated within job-related tasks and workplace rules (Graen and Scandura, 1987) that shape and drive formal firm strategies and performance throughout the hiring and talent management processes. The interorganizational networks are embedded and sustained into firms and are interconnected through networked employees, organizations and markets. Firm strategies and performance will decrease due to unobserved or invisible mismanagement in business practice and people. Clearly, “intentional human action and interaction causally produce strategic phenomena” (Abell et al., 2008, p. 492) that reconfigure a firm’s strategy and performance.

Few firms possess and hold all the resources needed to implement a strategy. In an ideal sense, the organization’s culture should encourage strategic thinking at every level of the organization. Employee networks could easily shape and constrain partnerships, value creation, and value capture in a business model. Many multi-business firms operating within a remote environment will evaluate the influence of certain developments such as political, social, economic, technological, and environmental factors on a specific business unit. However, no attention is paid to the influence of informal employee social network strategies that could easily reshape and redirect corporate perceived remote and industry environment developments. For instance, employees could easily influence the marketplace of firms within unproductive relationships that prevent partnering with other successful firms. Consequently, informal employee social network strategies impact market competition through power relationships among suppliers, buyers, entrants, substitutes and rivalry that influence current and future levels of prices, investment in the industry and firm performance and profitability. Furthermore, interorganizational open strategies require firms to develop their strategies collaboratively through a strategy process with other organizations. In addition, firms will formulate strategies for improving supply chain efficiency and decrease working capital within increasing market competition among networked relationships with organizations that must jointly share information to derive benefit from the interorganizational arrangement. Firms can develop differential advantage through employee networks due to changes in the competitive environment which in turn, lead toward sustainable competitive advantage because competitors will find them difficult to emulate (Clark and Collins, 2010). Since firms are dependent on employee social networks to drive and shape organizational strategies, employee social network management should be prioritized and integrated within a firm’s financial management. Supervisors must learn to manage subordinates learning and task performance not merely through data analysis, but rather in a socially connected world (Lee et al., 1999). The theoretical framework shows how employee social network strategies emerge and flow in a reciprocal manner from the organizational level to society at large. Knowing how to compete and cooperate (Rodriguez and Bharadwaj, 2017) from employee relationships to market competition is an imperative for managing organizational performance. Hence, proposition 4 suggests employee group entitativity within interorganizational networks decrease organizational performance.

## Discussion

### Managing Informal Employee Social Network Strategies

**FIGURE 4:**
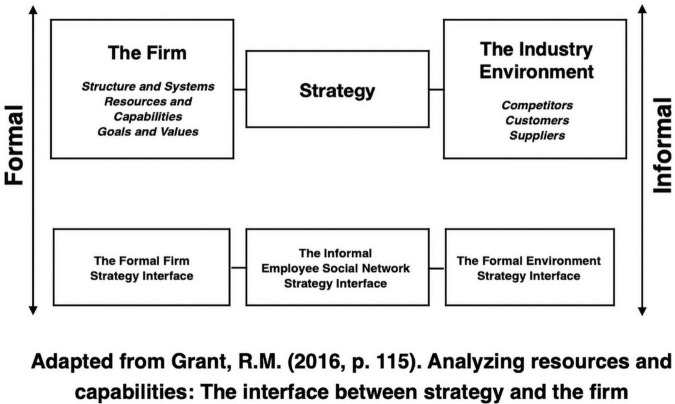
Analyzing informal employee social networks in formal strategy formulation.

The article demonstrates how and why employee social networks could influence firm strategies and organizational performance. The article’s research findings show some similarity to previous empirical evidence that reveal people will develop relationships with individuals that have similar interests and goals to themselves and negotiate control through informal freedom rather than formal absolute control and power (Burt, 1992). However, this research study does not focus on the value buried in structural holes through social capital (Burt, 2001). Rather, the article brings to light multilevel informal employee social dynamics that operate through simultaneously closed and open networks within firm strategizing activities. Group entitativity and value factors in the typology are mechanisms for self, shared, collective and relational interests that aggregate, develop and grow within informal employee socialization processes in the organization and externally with stakeholders and market competition. The underlying processes, mechanisms and alternate causal paths in social, relational, governance, network and organizational structures provide new insights and implications for firm strategies and organizational performance. Organizational strategy analysis pays attention to the external changing environment and market competition for potential impacts in business processes, organizational learning and growth, financial and stakeholder needs and firm specific resources and capabilities. Since routines and managerial decisions play a critical role in firm strategies and organizational performance, understanding the firm’s core competences through complex systems that evolve unpredictably is essential (Graen, 1976). For instance, Triggs and Leigh (2019) propose there is a growing body of research and experience that shows the “Chicago School’s faith in the ability of markets to self-correct and deliver competitive outcomes was misplaced” (p. 1). Therefore, firms must analyze formal firm strategies and formal environment strategies with informal employee social network strategies to improve firm strategies, resources, capabilities and the industry environment (Figure 4).

Since employee social network strategies are geographically dispersed, it is important for management to acknowledge network connections among market competitors, the firm’s employees and firm alliances for managing and improving firm strategies and organizational performance. Management should take into consideration informal employee social network strategies in the strategy making process for firm market positioning and when building and sustaining competitive advantage. It is common for strategists in the organization to fill the role of supervisor and staff in strategy. However, without considering informal employee social networks strategies there are unrealized strategies, missed opportunities and increasing risk. New strategies often require a new organizational culture, structure, and tasks that create a triggering event for accelerating informal employee invisible practices that span interorganizationally from the firm to market competition. Since competitive networks operating in a firm are an important and positive source of a firm’s competitive advantage (Wang and Gao, 2021), management should pay particular attention to how informal employee social networks obtain social control from firms. Likewise, Ramai et al. (2018) suggest some social networks can be socially non-conducive that raise questions over behavioral intensity, bullying, social distance, boundedness and exclusivity, and situations where members are mutually or reciprocally negative. This could happen through crossing closed homogenous network ties through open heterogenous network ties. Therefore, open strategies and open innovation will require critical examination of networked business professionals that operate and communicate in nebulous support networks across sectors, industries and communities worldwide.

Invisible deficiencies in organizations are never found on the risk register. Employees generally know and could predict the demise of their organization’s strategies well before organizational performance decreases, but the reasons are rarely discussed with managers, leaders and subordinates due to complex issues that are too sensitive to raise openly. Depicting employees as a tangible resource in an organization tends to mask the actual invisible individual and social activities involved (Cropanzano and Mitchell, 2005). Dark social networks (Grant, 2016) are not confined to the internet and cartels. Rather, dark networks are intertwined with positive or neutral social networks. Organizations will not benefit from strategy implementation if the employee small-scale actions are not examined. In addition, stakeholders in the external environment may be connected in routine small actions with employee social network strategies that could deceptively reconfigure firm decision-making and strategies, organizational performance, industry stakeholders and market competition.

### Implications for Firm Strategies and Organizational Performance

Research studies indicate knowledge spillovers and misappropriation are prevalent because of inter-firm competition, colocation, alliances, as well as employee mobility (Hamel et al., 1989; Shaver and Flyer, 2000; Berry, 2014; Inkpen et al., 2019). Unsurprisingly, permitting these type of actions and activities to foster and grow into and over other organizational goals and strategies generates systemic disruptions to firm strategies and organizational performance. Moreover, similar to formal firm strategies, informal employee social network strategies include a system of rules, sanctions and laws for individual and social cooperation within employee interactions that are inadequate and necessitate monitoring of self-interest (Peachey and Lerner, 1981; Liao et al., 2010). A key driver of improved individual performance may be cumulative experience (Neffke, 2019). Nevertheless, the value of experience can generate competency traps or core rigidities (Leonard-Barton, 1992) in strategy formulation through social network connections of small scale employee actions within day-to-day job routines in the organization that connect through employee-stakeholder social networks within market competition. Therefore, informal employee social network strategies require examination of “tight interlinkages between preferences, culture, and institutions” (Demeritt and Hoff, 2018, p. 2) for improving strategy formulation, implementation, organizational performance (Guiso et al., 2015), and market competition.

### Main Limitations and Future Research

A limitation of the theoretical framework is the particular culture and environmental context. Since cultures and characteristics in group entitativity may affect firm strategies and performance differently, future research could examine the network characteristics and cultures that are most likely to positively and negatively affect firm strategies and performance in their particular environmental context. Network size could be a limitation due to dense and sparse networks within differing organizational structures. Future research could conduct a comparative analysis of network size and organizational structures. A third limitation stems from network structures that predict similarity between attitudes and behaviors indirectly rather than directly (Burt, 1992) that could change over time. Longitudinal studies could help to shed more light on indirect changing group entitativity and competitive actions in employee social networks. In addition, future research could randomly select participants from the population to evaluate variation within firms’ departments, divisions and unit level network interactions to understand the static and “temporal dynamics” (Lehmann-Willenbrock and Allen, 2018, p. 326) that surround group entitativity within networked organizations and markets.

The theoretical framework could be operationalized quantitatively by using dynamic social interaction analysis techniques (Sekara et al., 2016) or social network analysis (Tichy et al., 1979; Marsden, 2005; Domínguez and Hollstein, 2014; Molina et al., 2014). Future research could investigate comparative micro-meso-macro links through the theoretical framework within different firm strategies across sectors and industries. Moreover, future research could compare small and large organizations to identify potential moderators of the relationship among employee networks, firm strategies and performance. Lastly, future research could investigate formal firm strategies in technology clusters (Speldekamp et al., 2020) within the theoretical framework to determine how dark networks could develop through employee social network strategies in organizational platforms and social media that connect with industry stakeholders and market competition to improve firm cybersecurity initiatives.

## Conclusion

Due to COVID-19, many firms are operating within networks of interconnected organizations and networks of individuals. Firms must evaluate informal employee social networks *with* firm strategies to improve strategy formulation, implementation and organizational performance. The propositions highlight the importance of informal employee social network strategies within individual and social competition and group entitativity could easily generate protective employee interorganizational network interactions that span across industry stakeholder connections and market competition, and in turn decrease organizational performance and market competition. The theoretical framework and key concepts, namely group entitativity, individual and social competition, and informal employee social networks provide a new interpretation that is distinct and bounded from other ways of theoretically framing firm strategies to improve strategy formulation, implementation and monitoring through corporate strategies, business unit strategies, functional strategies and operations level strategies. Overall, the article contributes to understanding how informal employee social network connections influence strategic activities, cybersecurity, diversity, equity and inclusion, and employee management.

## Data Availability Statement

The original contributions presented in the study are included in the article/supplementary material, further inquiries can be directed to the corresponding author.

## Author Contributions

MT formulated the idea, conducted the research, and wrote the manuscript.

## Conflict of Interest

The author declares that the research was conducted in the absence of any commercial or financial relationships that could be construed as a potential conflict of interest.

## Publisher’s Note

All claims expressed in this article are solely those of the authors and do not necessarily represent those of their affiliated organizations, or those of the publisher, the editors and the reviewers. Any product that may be evaluated in this article, or claim that may be made by its manufacturer, is not guaranteed or endorsed by the publisher.
